# Mysterious Outbreak of Acute Neurological Syndrome in Eluru, Andhra Pradesh, India: A Post-outbreak Geo-Spatial Analysis

**DOI:** 10.7759/cureus.31801

**Published:** 2022-11-22

**Authors:** Sumita Shankar, Limalemla Jamir, Rakesh Kakkar, Rajeev Aravindakshan, Mukesh Tripathi, Ravishankar Ayyanar, Mangayarkarasi V

**Affiliations:** 1 Department of Plastic and Reconstructive Surgery, Guntur Medical College, Guntur, IND; 2 Department of Community and Family Medicine, All India Institute of Medical Sciences (AIIMS) Guwahati, Changsari, IND; 3 Department of Community and Family Medicine, All India Institute of Medical Sciences (AIIMS) Bathinda, Bathinda, IND; 4 Department of Community and Family Medicine, All India Institute of Medical Sciences (AIIMS) Mangalagiri, Guntur, IND; 5 Department of Anesthesiology, All India Institute of Medical Sciences (AIIMS) Mangalagiri, Guntur, IND; 6 Law & Order, Andhra Pradesh Police Department, Mangalagiri, Guntur, IND; 7 Department of Microbiology, All India Institute of Medical Sciences (AIIMS) Mangalagiri, Guntur, IND

**Keywords:** environmental pollution, geographic information system, pesticides, poisoning, seizures, outbreak

## Abstract

Background: An outbreak characterized by sudden-onset seizures, loss of consciousness, and complete recovery within a few hours was reported from Eluru town in Andhra Pradesh on December 6, 2020. This study was conducted to assess the environmental correlates of the outbreak using geo-mapping tools.

Methods: A post-outbreak survey was conducted among affected cases in January-February, 2021. A house-to-house survey tool collected information on demographics, clinical profile, and environmental and psychological aspects (Impact of Event Scale). Geo-mapping and news media content analyses were done using QGIS and Atlas.ti software, respectively.

Results: A total of 394 cases were studied. The median (interquartile range [IQR]) age of the participants was 27 (17-39) years and comprised mostly male students. There was no clustering of cases within 48 hours of illness onset in the spatial analysis. Loss of consciousness was the first (50.7%) and the most common symptom. All cases were taken to a health facility and were discharged after a median duration of 48 minutes. COVID-19-related and environmental practices were not associated with the clinical manifestations. Awareness about pesticides was low. The outbreak reportedly had a psychological impact on 24.4% of the participants. The most common co-occurring themes in the news media analysis were water contamination and pesticides.

Conclusion: The geo-spatial analysis did not find case clustering or points of convergence during the incubation period. The geo-locations did not distribute around water bodies or suspected landmarks although news media projected water contamination and pesticides as probable causes of the outbreak.

## Introduction

In the first week of December 2020, the state of Andhra Pradesh attracted widespread attention when more than 500 cases of a sudden loss of consciousness, convulsions, giddiness, vomiting, and frothing occurred in a span of two days, in a fairly localized area of Eluru township in West Godavari District, Andhra Pradesh. Several technical and research institutes were promptly engaged by the government to investigate the outbreak [1]. The absence of fever led experts to suggest non-infectious causes of the outbreak [2]. Assumptions based on earlier studies ranged from non-psychogenic mass hysteria, poisoning due to heavy metals, or the more commonly available pesticides [3,4]. 

Additionally, due to the ongoing COVID-19 pandemic, there were speculations of a new variant of the severe acute respiratory syndrome coronavirus-2 and several media houses including international media arrived in Eluru to report about the outbreak [5]. Analysis of such media reports has helped in the early detection and surveillance of disease outbreaks [6]. Moreover, post-outbreak surveys are beneficial in providing an overview of the event in mitigation efforts and policy formulation [7].

Outbreak investigations are also greatly strengthened by using Geographic Information System (GIS) through integrative spatial data tools for visualization of case distribution and identification of location-specific etiologies, even if the causative agent becomes evident from biochemical or microbiological investigations [8].

The present post-outbreak survey was conducted to assess the environmental correlates of acute neurological illness outbreak among the affected cases in Eluru Mandal, West Godavari District using geo-mapping tools.

## Materials and methods

Study design and setting

The survey was conducted using a cross-sectional study design between January and February 2021. The study setting was Eluru municipality, which is the district headquarter of West Godavari District (WGD), Andhra Pradesh, India. As per Census 2011, the population of Eluru was 217,876. The Tammileru river and the Krishna and Godavari canals pass through the city before the river. The Eluru canal from the Krishna river empties into Kolleru lake near the city.

Study population

The study population comprised cases of acute neurological illness in Eluru town treated at the Eluru government hospital between the first and the second weeks of December 2020. This comprised approximately 623 cases. A line listing of these cases was done with demographic information, contact details, and clinical manifestations at hospital admission, for enrolment into the survey.

Sample estimation

The sample size was estimated using StatCalc software. To capture 40% true cases with typical presentation out of the reported 615 cases, assuming a confidence interval of 95%, allowable error of 4%, design effect of 1, and 25% loss of subjects, the sample size was estimated as 397 participants.

Convenient sampling was done to enroll available, less remotely located cases, considering logistical constraints due to the ongoing COVID-19 pandemic.

Data collection

Outbreak data were collected in three ways: a) the house-to-house survey, b) geographical mapping of movement of the cases prior to illness, and c) news reports of the outbreak.

a) House-to-house survey of the line-listed cases was done using a questionnaire generated in the local language, Telugu. Three attempts were made to contact each case. The questionnaire comprised demographic information and clinical profile of the participants such as symptoms, duration of hospital stay, symptom recurrence after discharge, past/family history, and co-morbidities. To assess the sources of possible exposure, portals of entry, and factors influencing spread, the participants were asked about diet and food hygiene, COVID-19-related practices, and environmental aspects, at personal and community levels.

Additionally, the Impact of Event Scale-Revised (IES-R) was administered to the participants. The IES-R is a 22-item self-report measure (for DSM-IV [Diagnostic and Statistical Manual of Mental Disorders, fourth edition]) that assesses subjective distress caused by traumatic events. Items correspond directly to 14 of the 17 DSM-IV symptoms of post-traumatic stress disorder (PTSD). Respondents were asked to identify the stressful life event such as what happened in Eluru and then to indicate how much they were distressed or bothered during the past seven days by each "difficulty" listed [9].

b) Geographical data: The cases were mapped according to the address and location within 48 hours of illness onset to identify any clustering in space, in addition to mapping recent changes in population due to social events or natural calamities. As per the Inter-governmental Forum on Chemical Safety, 48 hours is also a criterion for defining acute pesticide poisoning within which there is any illness or health effect from suspected or confirmed exposure [10].

Familial or locality-wise clustering of the cases was marked for environmental analysis. Drinking water supply, sanitation, animal/bird population in the area, agricultural activity, and any health-related activity were captured. Sources of these data were hospital records, official communication by state authorities, and government websites.

c) News reports: News reports of the outbreak, published in newspapers or websites of local, national, and international media in the English language, were obtained. A data extraction sheet was prepared in Microsoft Excel sheet. The contents were extracted by source, region of coverage, date, number of cases, areas affected, symptoms, etiology, laboratory/investigation findings, and any additional information.

Data analysis

An epidemic curve was created with the time of onset of illness as reported to the health authorities. Descriptive data analysis was done using SPSS software version 21.0 (IBM Corp, Armonk, NY) and data are presented as frequencies, percentages, mean (SD) and median (interquartile range [IQR]). QGIS software for GIS was used in the analysis. The initial case density mapping was done followed by spot maps for the available cases as per survey locations. Further, it was used to create overlays of impact assessment of the outbreak (using the percentage choropleths for affected proportions) and appropriately sized pie charts for each ward as per impacted/non-impacted numbers of persons. Atlas.ti software was used for content analysis wherein sentences that appeared in print media regarding the outbreak were marked as quotations and further coded. The codes were analyzed for co-occurrence and were cross-tabulated with regard to the most prevalent popular theory regarding the origin of the outbreak. A network diagram was created linking the themes thereof and a graphical overall view of the opinion space was generated regarding the outbreak.

## Results

**Figure 1:**
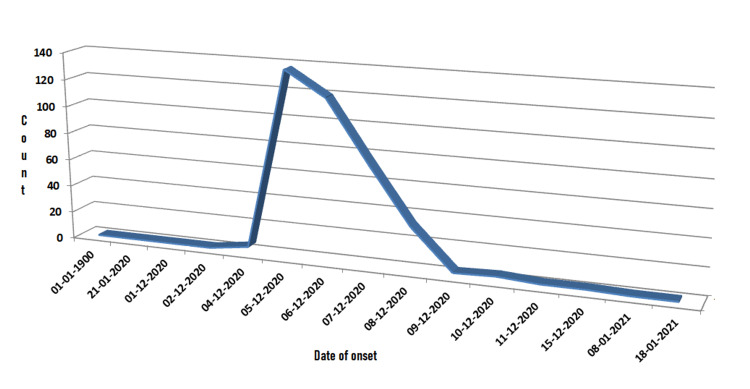
Epidemic curve as per survey.

The survey team was able to locate 394 cases using a line-listing epidemiology sheet by addresses provided during admission to the district hospital. The date of occurrence is presented in the epidemic curve, with the onset being abrupt and the highest number of cases recorded on midnight of 4th and 5th December 2020. Subsequently, the cases fell in a tapering manner (Figure 1).

**Figure 2:**
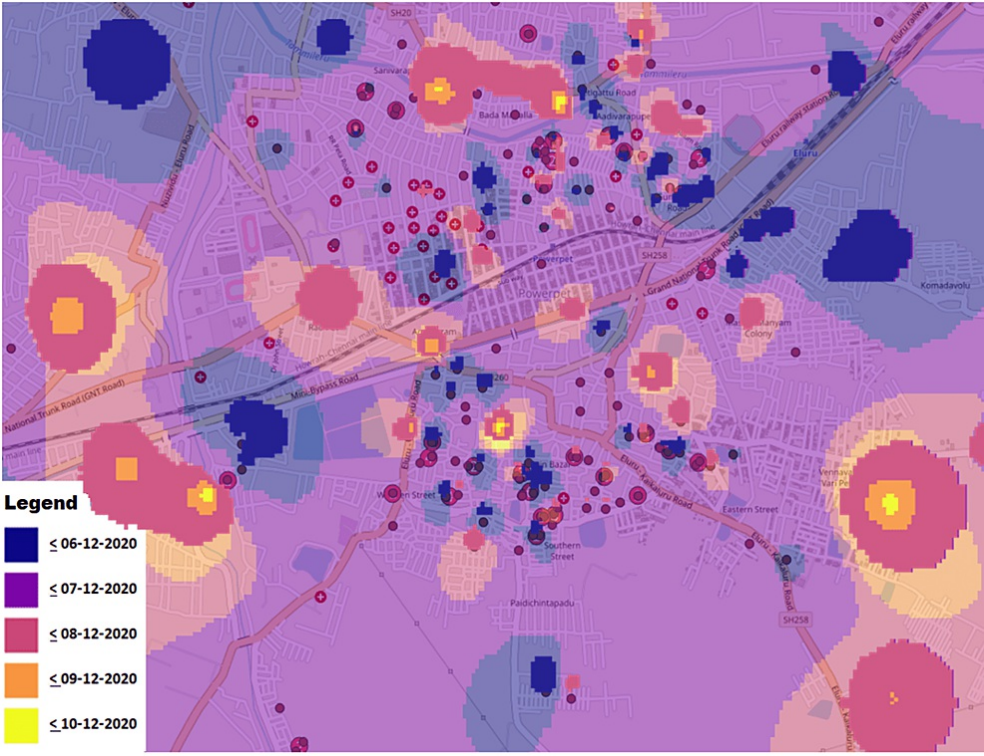
Timeline of clusters of cases (hospitals marked as (+))

Figure 2 depicts the possible early locales where the cases started and how the space-time clusters eventually terminated.

Demographic profile of the participants

**Figure 3:**
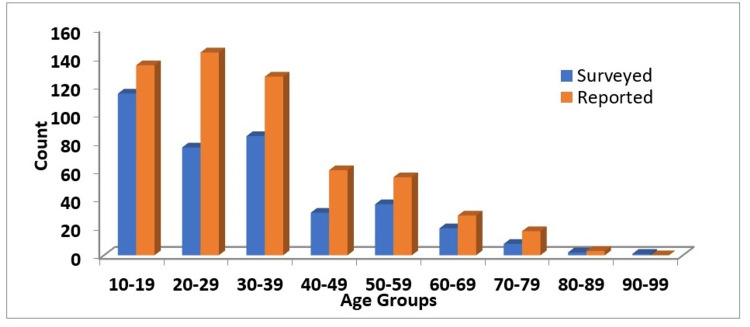
Age group (years) distribution of survey participants (n = 394; blue) versus total number as per hospital records (n = 615; red)

The age distribution of surveyed individuals corresponds with that of the total number of cases documented in the hospital or official records of the outbreak (Figure 3).

The median (IQR) age of the surveyed cases was 27 (17-39) years. Of this, 99 cases were less than 18 years of age. More than half (53.4%) of the participants were males and most of the cases in our survey were students (Table 1).

**Table 1:** Demographic profile of the cases *Median (IQR); ^#^Mean ± SD. IQR, interquartile range.

Variable		Number (%)
Age in years		27 (17-39)^*^
Sex		
	Male	210 (53.4)
	Female	183 (46.6)
Occupation		
	Daily wage laborer	63 (16.4)
	Private sector/self-employed	60 (15.7)
	Government sector	10 (2.6)
	Homemaker	87 (22.7)
	Student	139 (36.3)
	Others (including unemployed)	24 (6.4)
Family size		4.14 + 1.45^#^

Clinical profile of the cases

The first symptom that was also the most common symptom experienced by the participants was the loss of consciousness. Other symptoms were altered sensorium, headache, vomiting, and seizures (Table 2).

**Table 2:** Clinical profile of the cases *Multiple responses possible; ^†^Median (IQR).

Variable	Category	Number (%)	
First symptom that developed	Seizure	9	(1.8)
	Vomiting	31	(6.2)
	Headache	28	(5.6)
	Loss of consciousness	252	(50.7)
	Altered sensorium	150	(30.2)
	Paralysis	19	(3.8)
	Fever	3	(0.6)
	Other(s)	5	(1.0)
Symptoms during the illness^*^	Seizure	28	(4.3)
	Vomiting	76	(11.6)
	Headache	84	(12.9)
	Loss of consciousness	234	(35.8)
	Altered sensorium	186	(28.5)
	Paralysis	16	(2.5)
	Fever	9	(1.4)
	Other(s)	20	(3.1)
Duration between illness onset and treatment in health facility^†^		30 (21-30) minutes	
Admitted in a hospital	Yes	385	(98.2)
	No	7	(1.8)
Duration of stay in hospital^†^		48 (24-72) minutes	
Recurrence of symptoms after discharge	Yes	75	(19.2)
	No	315	(80.8)
Similar episodes in the past	Yes	26	(06.6)
	No	367	(93.4)
Similar illness in family member(s)	Yes	32	(8.1)
	No	361	(91.9)
Co-morbidities	Present	85	(21.6)
	Absent	309	(78.4)
Type of co-morbidities^*^	Epilepsy	11	(12.8)
	Hypertension	28	(32.6)
	Diabetes mellitus	28	(32.6)
	Heart disease	8	(9.3)
	Kidney disease	9	(10.5)
	Lung disease	2	(2.3)
	i. Tuberculosis	11	(12.8)
	Others	9	(10.5)
Tobacco intake	Never taken	351 (89.1)	
	Currently taking	35 (8.9)	
	Taken in the past	8 (2.0)	
Unusual food intake in the last one week before illness	Yes	7	(1.8)
	No	386	(98.0)
	Cannot remember	1	(0.3)
Travel in the last two weeks before illness	Yes	2	(0.5)
	No	391	(99.5)
History of contact with COVID-19 case	Yes	17	(4.3)
	No	377	(95.7)
Documented investigation reports available	Yes	271	(68.8)
	No	123	(31.2)

A total of 11 participants residing in different localities had a history of epilepsy. All participants were taken to a health facility and were discharged after a median duration of 48 minutes. About 271 (68.8%) participants had investigation reports with them. Biochemical parameters of the cases such as complete blood profile, serum urea, serum creatinine, random blood sugar, and serum bilirubin were normal. All cases had tested negative for infectious diseases, namely COVID-19, hepatitis B, typhoid, and malaria. Other investigations done were ECG, chest x-ray, electroencephalogram (EEG), and CT brain, results of which are out of the purview of this post-outbreak survey.

Diet and hygiene-related practices of the cases

Most (85.3%) of the participants consumed a vegetarian diet and practiced food hygiene (Table 3). However, there were cases who did not practice washing fruits or vegetables before consumption or washed their hands with soap and water before meals. Fifty participants did not wear face masks in the last 30 days.

**Table 3:** Diet and hygiene-related practices of the cases

Variable	Category	Number (%)
Type of diet taken	Vegetarian	336 (85.3)
	Non-vegetarian	58 (14.7)
Fruits or vegetables washed for at least 2 minutes before eating or cooking	Never	55 (14.0)
	Sometimes	5 (1.3)
	Most of the time	1 (0.3)
	Always	333 (84.5)
Hands washed with soap and water before meals in the last one month before illness	Never	56 (14.2)
	Sometimes	5 (1.3)
	Most of the time	0 (0.0)
	Always	333 (84.5)
Wearing of face mask when outdoors in the last one month before illness	Never	50 (12.7)
	Sometimes	2 (0.5)
	Most of the time	1 (0.3)
	Always	341 (86.5)

Environmental aspects assessed in the survey

**Figure 4:**
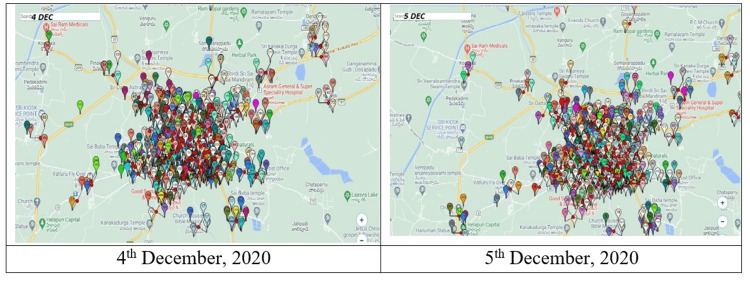
Locations of the cases 48 hours prior to the day of onset (courtesy: AP Police Department)

Figure 4 shows the antecedents of the cases by location, within 48 hours of illness onset, which did not show any common meeting points in space or time. The environmental aspects assessed in the survey are presented in Table 4.

**Table 4:** Environmental aspects assessed in the survey

Variable	Category	Number (%)
Type of water supply	Municipal supply	368 (95.3)
	Borewell	5 (1.3)
	Open well	6 (1.6)
	Others	7 (1.8)
Building material of walls of the house	Cement	380 (96.4)
	Brick only	6 (1.5)
	Mud	8 (2.0)
Recent remodeling or repairs of the house	Yes	11 (2.8)
	No	382 (97.2)
History of pica (paint, soil, chalk, raw rice, etc.)	Yes	18 (4.6)
	No	376 (95.4)
Pets/animals owned by the household	Yes	43 (10.9)
	No	351 (89.1)
Type of pets/animals owned	Cat	2 (7.1)
	Dog	24 (85.7)
	Poultry	2 (7.1)
Involved in applying pesticides to crops in the last six months	Yes	1 (0.3)
	No	393 (99.7)
Protective measures taken before and after application of pesticides	Yes	2 (0.5)
	No	391 (99.5)
Hands washed with soap in order to eat, drink, or smoke, in between or after pesticide application	Yes	57 (14.5)
	No	335 (85.2)
	Don’t know	1 (0.3)
Trained on the health risks of pesticides	Yes	1 (0.3)
	No	392 (99.5)
	Don’t know	1 (0.3)
Spraying activity in the locality against COVID-19	Yes	96 (24.4)
	No	291 (74.0)
	Don’t know	6 (1.5)
Spraying/fogging activity in the locality against malaria	Yes	24 (6.1)
	No	322 (81.7)
	Don’t know	48 (12.2)

Psychological impact among cases of the outbreak

**Figure 5:**
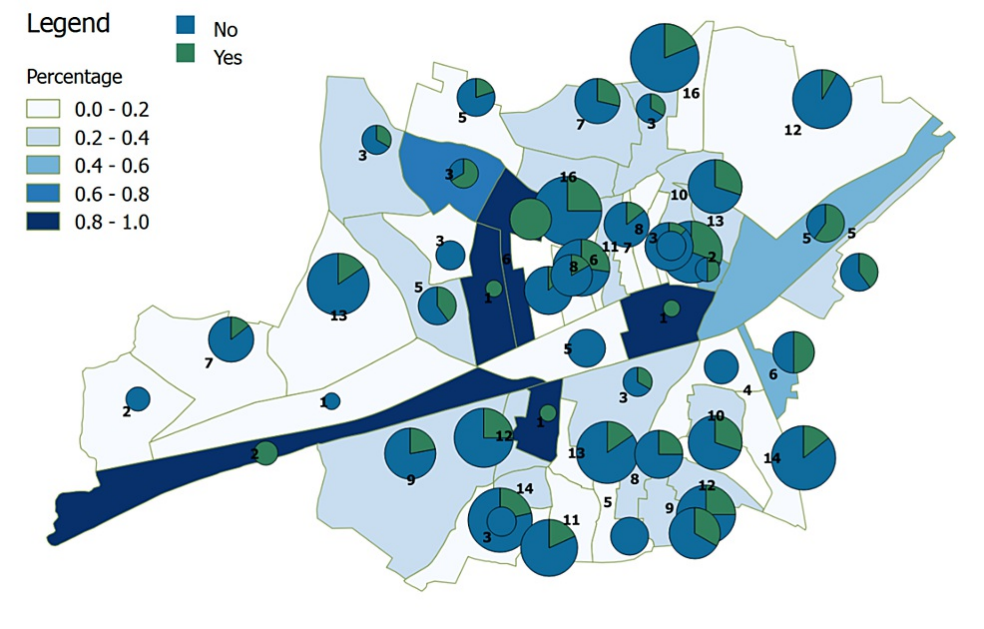
Psychological impact among cases in the surveyed area as per GIS locations GIS, Geographic Information System.

A total of 96 (24.4%) cases responded that the outbreak had a negative psychological impact on them. The number of survey respondents per ward and the impact it had on them are presented in Figure 5.

Content analysis of print media reports on the outbreak

**Figure 6:**
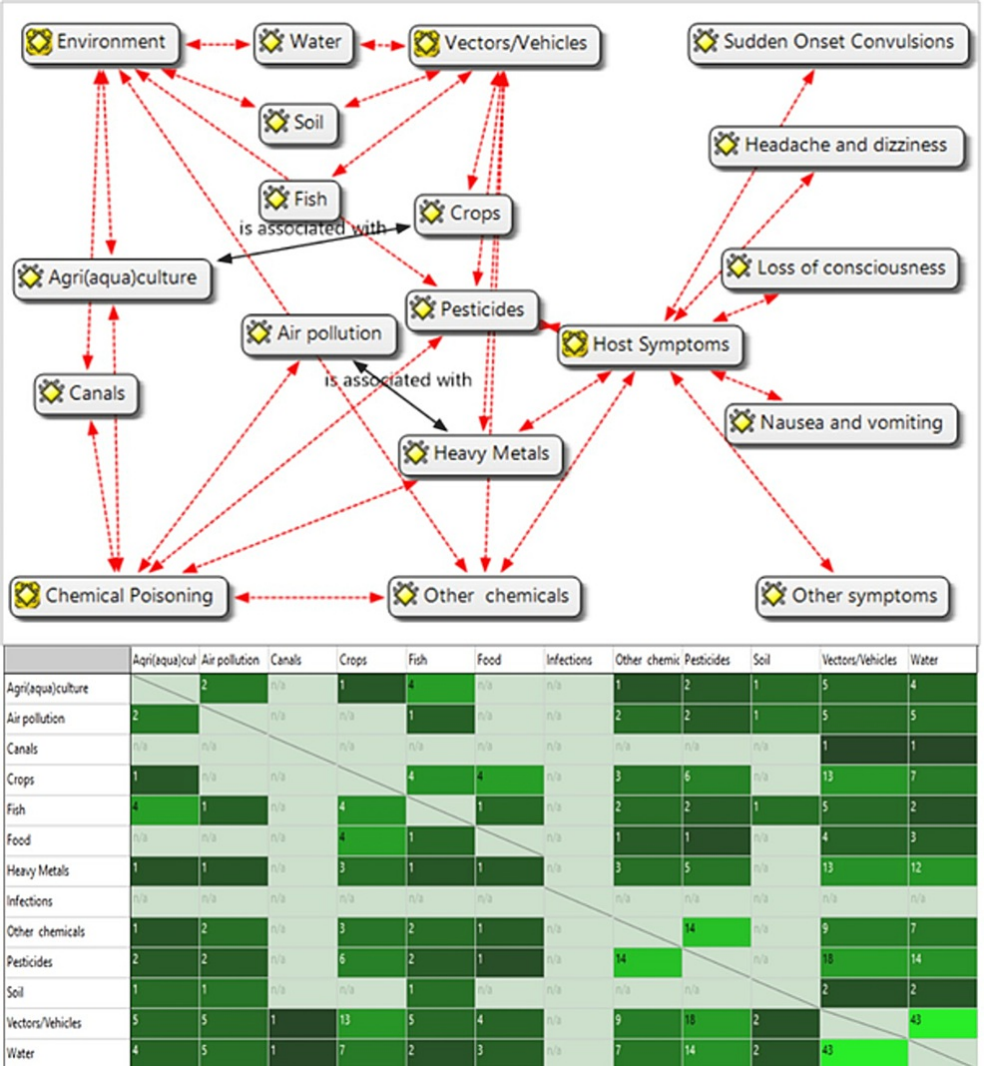
Content analysis and code co-occurrence of print media reports on the outbreak

A total of 65 print media reports on the outbreak in English, from 06.12.2020 to 22.03.2021, were analyzed. This comprised newspapers and online web portals including local, national, and international press such as *Deccan Chronicle, The Hans India, Times of India, New Indian Express, The Hindu, The Quint, India Today, Business Today, news18, Washington Post, Associated Press, TIME, The Guardian*, etc. While most news reports were based on statements made by government officials or the investigating agencies, some were first-hand information about the cases. The most common symptoms mentioned were sudden onset of convulsions, loss of consciousness, headache, and nausea/vomiting. The likely causes mentioned were contaminated water or food, laden with chemicals such as pesticides, or heavy metals as found in the water/vegetable and blood samples, respectively. These were attributed to the environmental risk factors present in the area such as poor sanitation, unregulated industrial waste, and increasing use of harmful chemicals for agricultural purposes. The region being flood-prone was also cited as a potential risk factor for such a calamity. The findings are summarized in Figure 6. The most common co-occurring relevant themes were water and pesticides.

## Discussion

The survey located more than 60% of the cases of the outbreak of acute neurological illness as per the contact details provided at the time of admission to the emergency department of Eluru government hospital. The response rate corroborates with the finding of contact numbers belonging to the accompanying person(s) and not the case per se, and some of the cases having relocated, at least temporarily, due to the prevailing outbreak situation as observed in previous post-outbreak surveys [7].

The peak of the outbreak occurred over the first weekend of December 2020 and the majority of the cases were males and students. Since this demographic was congruent with those of the official government reports, the possibility of exposure while outdoors over the weekend was explored through GIS spatial mapping in the present study. However, no significant clustering of cases within 48 hours prior to illness onset was found.

The first symptom, also the most common clinical feature reported by the participants, was the loss of consciousness. The most common causes of such transient loss of consciousness are epileptic seizures, syncope, or psychogenic non-epileptic seizures [11]. However, such cases often confound with normal inter-episodic investigations and non-specific abnormalities [12]. In the present study, the manifestations of post-ictal confusion or altered sensorium, amnesia, tongue bite, and headache were more suggestive of seizure than syncope or psychogenic non-epileptic seizure. About 19% of the participants had a recurrence of symptoms after discharge which warrants close monitoring, as new-onset seizures can progress to epilepsy [13].

The causes of new-onset seizures are mainly due to acute insults to the brain by noxious or infectious agents in the environment [13]. In the present study, the majority of the participants consumed the usual diet in the week preceding the illness and their symptoms were largely of non-infectious origin. This was substantiated by the negative test reports of the cases for common infectious diseases and COVID-19. Although inhalational poisoning was a possibility, the lack of respiratory manifestations and the absence of dead animals in the vicinity were against an assumption of inhaled environmental poison in the outbreak causation pathway.

Poisoning by pesticides such as organophosphates, organochlorines, and pyrethroids suspected in the outbreak are known causes of accidental, suicidal, and homicidal poisoning in India [14]. The lower case fatality in the current outbreak is suggestive of organochlorine or pyrethroid poisoning [15]. Moreover, convulsions are relatively rare in organophosphate poisoning, which is characterized by excessive salivation, sweating, and pin point pupil, often with poor prognosis [16]. The latest report of the outbreak by the Indian Council of Medical Research that conducted detailed laboratory analyses reported "relatively" high concentrations of triazophos pesticide in municipal water of affected households; however, the source of contamination was not identified [17].

A high prevalence of toxigenic heavy metals in groundwater, soil, house paints, etc. has also been reported in the country [18,19]. However, in the present study, the clinical course and spatial distribution of the outbreak do not conform to typical heavy metal poisoning which is largely characterized by the chronicity of exposure.

Nevertheless, several environmental health hazards such as poor quality of drinking water and contamination of treated surface water have been reported in Eluru [20,21]. Contamination of the Pampula Cheruvu reservoir, which supplies municipal water from the Krishna river through the Eluru canal system, was also implicated in the outbreak. The Eluru canal is reportedly contaminated with industrial effluents while passing through major commercial hubs. As the state of Andhra Pradesh is rapidly striving toward industrial and agricultural advancement, chemical incidents and crimes are not uncommon [22]. The current outbreak is also the second in illnesses of “unknown etiology” in the state, after "Uddanam" nephropathy, a chronic kidney disease considered endemic in the Srikakulam district [23]. Incidentally, four weeks after the outbreak, a nearby village supplied by the same river irrigation system reported 29 cases of similar illness with "unknown" etiology [24]. 

Additionally, Eluru Mandal is prone to floods and cyclones. For the past three months preceding the outbreak, Eluru was listed on disaster warning systems for floods from the overflowing Tammileru river and cyclonic rain from cyclone Nivar [25,26]. As floods are known to trigger chemical release from farmlands, roads, and industries [27], the possibility of this runoff contaminating crops, water supplies, and deposition on habitation while receding cannot be overlooked.

The analysis of news reports corroborated correctly with the symptoms stated by the study participants. Most news captions included the words “Mystery illness” adding further intrigue. The majority of the reports stated that diagnostic laboratory reports were non-specific and inconclusive. The main agents attributed to the outbreak were heavy metals, pesticides such as organophosphorus or organochlorine compounds, or insecticides. Major sources of exposure were stated as contaminated water, fish, or food staples. Incidentally, preliminary inputs by the cases while admitted to the hospital had described water-related issues such as a change in color and odor of water supplied, before the symptoms started. In the present study, however, responses related to water supply and other environmental variables only vaguely pointed to the possible source of illness.

Additionally, the psychological impact of a sudden, unknown illness on a mass scale is possibly higher, as compared to that caused by a known etiology. In our survey, 96 participants reported being psychologically affected by the outbreak although IEC cutoff scores were not met by any of them. Long-term somatic and psychological effects have been reported among survivors of natural disasters and criminal attacks in public spaces [28,29]. Psychological stress is known to persist in communities with chronic environmental contamination due to real or perceived health risks and inaction by authorities [30]. Hence, it is advisable to routinely monitor cases of the current outbreak and provide professional support as required.

Strengths of this study included the use of phone-based GIS tracking in defining case density and impact by wards and pinpointing early spots in the evolution of the outbreak. Another utility of GIS was by tracing the cases back in the potential incubation period to identify possible space-time clustering. However, none of these proved conclusive in terms of identifying the overlaps of agent-host-environment in this outbreak, besides the fact that the outbreak resulted in an aftershock along the canal system pointed to a general ecological cause rather than a definitive geographic location.

Limitations of the survey include the relatively lower coverage rate than estimated. However, this is an established limitation of post-outbreak surveys. Our survey objectives of a comprehensive post-outbreak analysis were achieved. Self-report of practices and the psychological impact of the outbreak could have affected the inferences. Also, the raw data had to be cleaned extensively for meaningful interpretations, due to navigation issues for the GIS mapping. Detailed geo-spatial assessment of human-environment interaction and symptom-specific recurrence was not done in the present survey.

## Conclusions

In conclusion, there was no confluence of affected persons within 48 hours of illness onset in the spatial analysis. The tracking of affected people did not reveal any common points where the patients converged during the incubation period if any. All participants were taken to a health facility and were discharged after a median duration of 48 minutes. Awareness about pesticides was low. News media projected water and pesticides as probable causes of the outbreak. However, the locations did not distribute around water bodies or such landmarks. The start of the outbreak was not localized to any particular hotspot, rather it was all around the township.

Although the probable etiology of the outbreak is now known, given the lack of understanding of the source, system preparedness and readiness can avert future incidents. Investing in safe, non-toxic, and novel technologies will benefit both the industry and the agriculture sectors in the long run. Upgradation in healthcare technologies to handle environmental toxins, poison management systems, and training of health personnel are the need of the hour. Intersectoral research and development projects would enable a better understanding and management of natural and man-made disasters.
